# Prevalence of *Plasmodium falciparum* Parasitaemia and Its Correlation with Haematological Parameters among HIV-Positive Individuals in Nigeria

**DOI:** 10.1155/2014/161284

**Published:** 2014-03-04

**Authors:** Olusola Ojurongbe, Oluwatoyin Adeola Oyeniran, Oyebode Armstrong Terry Alli, Sunday Samuel Taiwo, Taiwo Adetola Ojurongbe, Adekunle Olugbenga Olowe, Oluyinka Oladele Opaleye, Oluwaseyi Adegboyega Adeyeba

**Affiliations:** ^1^Department of Medical Microbiology & Parasitology, Ladoke Akintola University of Technology, PMB 4400, Osogbo, Nigeria; ^2^Department of Biomedical Science, Ladoke Akintola University of Technology, PMB 4400, Osogbo, Nigeria; ^3^Department of Mathematical and Physical Sciences, Osun State University, PMB 4494, Osogbo, Nigeria

## Abstract

Malaria and HIV are the two most important health challenges of our time. Haematologic abnormalities are features in *Plasmodium falciparum* infection, and anaemia is a well-known outcome. The prevalence and haematological impact of *P. falciparum* parasitaemia were determined among HIV-infected individuals in Nigeria. Parasite detection was carried out using microscopy and Polymerase Chain Reaction (PCR). Haemoglobin concentration was determined using an automated machine while CD4+ T-cells count was analyzed using flow cytometer. Thirty-seven (18.5%) out of the 200 HIV individuals enrolled had malaria parasites detected in their blood. All the positive cases were detected by PCR while only 20 (10%) were detected by thick blood microscopy. The mean haemoglobin concentration and packed cell volume (PCV) of HIV individuals with malaria parasitaemia were lower compared to those without malaria parasitaemia but the difference was not statistically significant. Also no significant difference was observed in malaria positivity in respect to sex and mean CD4+ cell count. The study highlights the effects of *P. falciparum* parasitaemia on the haematologic and immune components of HIV individuals.

## 1. Background

The two greatest health challenges facing Africa today are human immunodeficiency virus (HIV) infection and malaria [1, 2]. Worldwide, 225 million malaria cases were estimated with 176 million in the African region and 49 million elsewhere and 781,000 deaths in 2009 [3]. About 33 million people are currently living with HIV worldwide, with 22.5 million cases in sub-Saharan Africa where it is now the leading cause of death in the region. Nigeria has over four million persons living with HIV/AIDS with the north-central region harboring the highest HIV infection levels in the country [4].

There have been some controversies on the incidence of malaria among HIV individuals. While some studies reported high incidence of malaria in HIV individuals in malaria endemic areas [5] others have reported otherwise [6, 7]. The well-established fact is that malaria is a powerful stimulator of the immune system and subjects exposed frequently to malaria have enhanced serum levels of immunoglobulins and an accelerated rate of IgG turnover [5]. Therefore, malaria might have an adverse effect on HIV infection both by stimulating the T-cell turnover and by impairing T-cell cytotoxic function [8]. *Plasmodium* parasitaemia differs in instances of asymptomatic and clinical malaria, and the degree of parasitaemia may influence the pathological and biochemical presentations of individuals presenting with either of these conditions [9, 10]. Reports have shown that in most clinical cases of malaria, anaemia is a prominent factor, which is caused by destruction of infected blood cells by the reticuloendothelial system and haemolysis of infected cells [11, 12].

Different PCR-based assays have been used in diagnosis and as reference or standard for the assessment of the sensitivity and specificity of microscopy and commonly deployed rapid diagnostic tests. PCR assays though used mainly for research purposes may indeed be of clinical value in some selected situations [13–15]. PCR that targets the stevor gene, a multiple copy sequence of the subtelomeric variable open reading frame of *P. falciparum*, has been shown to be useful in the detection or measurement of very low parasitaemia [16, 17]. The stevor gene PCR was shown to be more sensitive than genus and species-specific PCR, which was designed to amplify portions of the sequences coding for the small subunit ribosomal RNA (SSUrRNA), and the highly polymorphic merozoite surface (MSP) antigens of *P. falciparum*, which is useful in determining genetic variability of parasite isolates [18].

This study therefore determines the prevalence of asymptomatic *falciparum* malaria in HIV patients using both microscopy and stevor PCR. The study also looked at the effect of age, sex, CD4+ T-cell count, and haemoglobin level on prevalence *falciparum* parasiteamia.

## 2. Methods

### 2.1. Study Group

Two hundred HIV-seropositive confirmed individuals who came for normal routine medical checkup at the institute of human virology of Nigeria (IHVN), Ladoke Akintola University of Technology (LAUTECH) Teaching Hospital Osogbo, Osun State Nigeria, were recruited into the study. Their HIV status was confirmed using Determine HIV1/2 kit (Abbott Diagnostic Division, Hoofddorp, The Netherlands) followed by Unigold or stat pak concurrently according to serial algorithm of the Federal Government of Nigeria [19]. Those who gave formal informed consent and were willing to be tested for malaria parasitaemia were recruited into the study. Ethical approval was obtained from the ethical committee LAUTECH Teaching Hospital, Osogbo, Osun state, Nigeria.

### 2.2. Blood Collection and Analysis

5 mLs of blood was aseptically drawn by venipuncture into ethylene diaminetetra-acetic acid anticoagulant bottles. The blood was used for the assessment of thin and thick films for malaria parasitemia, estimation of haematological parameters, and CD4+ T-cell count estimation. Two drops of blood were also spotted on filter paper, air dried, placed in individual sealed plastic bags, and kept at room temperature for DNA extraction and PCR analysis. The haemoglobin concentration of the collected blood samples was determined using an autoanalyser-Sysmex KX-21 (Sysmex Corporation, Kobe, Japan). Blood was collected into heparinized capillary tube up to two-third of its length for packed cell volume (PCV) estimation. Anaemia was defined according to the WHO criteria [20]. For males anaemia was defined as haemoglobin concentration (Hb) less than 13 g/dL, while for females, the value is less than 12 g/dL. CD4+ T-cell count was analyzed using a two-colour single platform flow cytometer (Partec Cyflow) according to the manufacturer's instructions.

### 2.3. DNA Extraction and PCR

DNA was extracted from the dried blood spots on filter paper using the Ultra Clean Blood Spin Kit (MO BIO Laboratory, Inc., Loker Ave West, Carlsbad) following the manufacturer's protocol. 100 *μ*L of distilled water was used to elute the DNA and stored at −20°C until further analysis.

Stevor gene PCR protocol was carried out as previously described [18]. Briefly, the PCR assay included a primary and nested reaction to enhance specificity. Amplification was performed using a GeneAmp PCR System 9700 thermal cycler (Biotron, Göttingen, Germany). Primary amplification was performed with reaction mixture of 50 *μ*L containing 5.0 *μ*L ×10 reaction buffer, 200 *μ*M of dNTPs, 1.25 units of Taq DNA polymerase, 0.4 pM of each primer (P5, P18, P19, and P20), and 5.0 *μ*L of DNA extract. The PCR programme included denaturation at 93°C for 3 minutes followed by 25 cycles of 30 seconds at 93°C, 50 seconds at 50°C, and 30 seconds at 72°C and a final extension period of 3 minutes at 72°C. Two microlitres of the first-round PCR product were used in the second round amplification which was performed in a reaction mixture of 50 *μ*L containing 5.0 *μ*L ×10 reaction buffer, 200 *μ*M of dNTPs, 1.25 units of Taq DNA polymerase, and 0.4 pM of each primer (P17 and P24). Conditions for the nested reaction included denaturation at 93°C for 3 seconds followed by 25 cycles of 30 seconds at 93°C, 50 seconds at 55°C, and 30 seconds at 72°C and a final extension period of 3 minutes at 72°C. DNA extracted from laboratory adapted *P. falciparum* (FCR-3 strain) was used as positive control and water as negative control. PCR products were subjected to electrophoresis on 2% agarose gels and visualized using Syngene gel documentation system (Syngene, Cambrige, UK) after staining with ethidium bromide. The primer sequences for the stevor PCR were as previously described [18].

### 2.4. Data Analysis

The data were analyzed using SPSS version 16.0 statistical software. Analysis of variance (ANOVA) and regression analysis were used to investigate the relationship between the variables. Chi-square (*χ* ^2^) was also used to test the association between categorical variables. All tests were carried out using the 5% level of significance.

## 3. Results

Among the two hundred HIV-positive patients enrolled in this study (male : female, 48 : 152), 20 (10%) were positive for malaria by microscopy while 37 (18.5%) were positive by stevor gene PCR. The mean age of the studied individuals was 36.24 (SD ± 10.10) years. Of the overall infected individuals, 27 (72.97%) were females while 10 (27.03%) were males. The mean haemoglobin concentration and packed cell volume (PCV) of the HIV-positive individuals were 10.85 g/dL (SD ± 1.89) and 32.53% (SD ± 4.41), respectively, while the mean CD4+ count was 391.31 (SD ± 237.14). The breakdown of the general characteristics of the study population is shown in Table 1.

Table 2 shows the comparison between malaria positive and negative HIV individuals based on age, sex, PCV, haemoglobin, and CD4+ T-cell counts. The mean age of malaria negative HIV individuals (36.78 years SD ± 10.38) was higher than their positive (33.92 years SD ± 9.34) counterparts and the difference was not statistically significant (*P* = 0.12). Similarly the mean haemoglobin level of malaria positive (10.86 g/dL SD ± 1.87) HIV individuals was lower in comparison to their malaria negative (10.80 g/dL SD ± 1.71) individuals and the difference was not statistically significant (*P* = 0.87). Also for the other parameters (sex and PCV) no significant difference was observed (Table 2).

Prevalence of malaria parasites, with respect to sex and age, and parasite density are shown in Table 3. The age group of 31–40 years had the highest prevalence (46.0%; 17/200) of malaria parasitaemia but was not statistically significant. Also no significant difference was observed in age group and sex with respect to parasite density (Table 3).

The comparison between the prevalence of malaria parasitaemia and CD4+ T-cell count is shown in Table 4. The highest prevalence of malaria parasitaemia (31.5%) was observed among the group that had the highest CD4+ T-cell count and the difference was statistically significant.

## 4. Discussion

HIV-infected patients are at higher risk of opportunistic infections including malaria because of their weakened immune systems. Sub-Saharan Africa carries a high burden of both malaria and HIV diseases thus coinfection is common in many areas [20]. A prevalence of 18.5% (37/200) of falciparum malaria parasitaemia was observed among HIV-infected individuals in this study and only 12% of this prevalence was detected by microscopy. This relatively low prevalence may be due to daily use of cotrimoxazole prophylaxis that is recommended for all symptomatic and asymptomatic adults and children living with HIV in Africa which has been documented to offer protection against malaria among HIV individuals [21].

The mean PCV and haemoglobin concentration of the malaria infected HIV individuals were 32.53% and 10.86 g/dL, respectively. The standard value of PCV is 47 ± 5% for men and 42 ± 5% for women while those of haemoglobin concentrations were 13 ± 2 g/dL for men and 12 ± 2 g/dL for women. While the haemoglobin concentration fell within the normal range, the PCV of the studied HIV population was not within the normal range, indicating that the population may be anaemic. Studies have shown that hemoglobin and PCV screening tests may not be comparable in detecting anemia in the same population especially in studies using venous blood, which may probably explain the differences seen in this study [22, 23]. Anaemia is one of the haematological complications of HIV-infected patients and it is recognized as an independent risk factor for early death among HIV/AIDS-infected individuals. Anaemia remains the prognostic marker of future progression or death, independent of CD4+ count and viral load among HIV individuals [24]. In this study, the presence of falciparum malaria parasitaemia was not associated with anaemia. This observation could be attributed to the fact that all the HIV individuals with malaria in this study had a very low density and/or submicroscopic *P. falciparum*. These low density parasites were unlikely to have contributed significantly to the presence of anemia in this study population. Clinical *falciparum* malaria is well documented to be associated with a reduction in haemoglobin levels frequently leading to anaemia [12, 25].

As expected stevor PCR was found to be more sensitive for detecting *P. falciparum* parasitaemia compared to microscopy. Different PCR-based methods have been consistently shown to be a powerful tool for malaria diagnosis [26, 27]. Stevor gene-based PCR has been shown to be at a vantage position since it is designed to amplify fragments of a multicopy gene (between 30 to 40 copies per haploid genome) compared to microscopy and even other PCR target genes [18]. The stevor gene-PCR has been reported to be at least 100-fold more sensitive than other PCR assays [16]. This observation is surely a renew call for the development and employment of a more sensitive tool for routine diagnosis of malaria. To date, routine microscopic examination of Giemsa-stained blood smears remains the gold standard for malaria diagnosis. The major advantage of the Giemsa-stained microscopy over other methods is that it allows for quantification of parasitaemia which is lacking in PCR and other available rapid diagnostic methods. On the other hand PCR-based methods are more sensitive in the detection of low levels of parasitaemia in asymptomatic infections and possess the ability to detect mixed Plasmodium infections. PCR might therefore be needed to complement blood smear microscopy and rapid diagnostic methods in routine diagnosis of malaria infection.

In our study, highest prevalence of malaria parasitaemia was observed among HIV-positive group that had the higher CD4+ T-cells count. Contrary to our observation, many studies have reported that malaria coinfection with HIV decreases CD4+ T cells counts [6, 28]. Although it has also been reported by other authors that malaria appears not to have any direct impact on the level of depletion of CD4+ T-lymphocytes in the HIV-infected subjects [29, 30]. Many other factors like nutrition and intake of nutrients could contribute to haematological parameters of HIV individuals [29, 31]. However, studies have shown that rates of malaria episode in HIV-infected individuals increase with falling CD4 T-cell count. In general, HIV-infected adults are more likely to develop clinical malaria and this risk becomes more pronounced with advancing immunosuppression such that at CD4 T-cell count below 200 cells/*μ*L (AIDS) individuals will experience increased incidence of malaria infection [32, 33]. HIV infection has been associated with a reduction in the absolute numbers of CD4+ T-lymphocyte count and untreated malaria infection appears to further reduce the CD4+ T-cells count [6, 32]. A prospective study found significant increases in mean CD4 counts (297 to 447/*μ*L) and decreases in the proportion of patients with a CD4 T-cell count less than 200/*μ*L (28.7% to 13.2%) after successful malaria treatment in HIV-infected individuals [34].

## 5. Conclusion

In conclusion, our study observed that 18.5% of the HIV individuals have *P. falciparum* parasitaemia without any direct contribution to anaemia. The need for constant screening and treatment of HIV individuals living in malaria endemic area for malaria parasitaemia is therefore suggested. Improved diagnostic technique is also suggested in other to effectively detect these parasites.

## Figures and Tables

**Table 1:** Enrollment data of the HIV patients attending IHVN Clinic recruited into the study.

Number of subjects examined	200
Mean age (years) ± SD	36.24 ± 10.01
Sex (male/female)	48/152
Mean haemoglobin	10.85 ± 1.88
Mean packed cell volume (%)	32.53 ± 4.41
Mean CD4+ count(cells)	391.31 ± 237.14
Number of positive by microscopy (%)	
(i) *P. falciparum *	20 (10)
(ii) *P. malariae *	2
*P. falciparum* positive by stevor PCR (%)	37 (18.5%)

**Table 2:** Comparison between malaria positive and negative HIV patients attending IHVN Clinic recruited into the study.

Characteristics	Malaria positive (PCR) *N* = 37	Malaria negative *N* = 163	*P* value
Mean age (years) ± SD	33.92 ± 8.55	36.78 ± 10.38	0.12
Sex: male/Female	10 : 27	38 : 125	0.38
Mean haemoglobin ± SD	10.86 ± 1.87	10.80 ± 1.71	0.87
Mean PCV (%) ± SD	32.71 ± 4.86	32.58 ± 4.28	0.88
Mean CD4^+^ cell ± SD	390.1 ± 218.41	391.6 ± 241.82	0.97

Significant at <0.005.

**Table 3:** Level of parasiteamia with respect to sex and age grouping.

Parameters	Examined number (%)	Positive number (%)	Parasite densities (parasite/*μ*L) (%)			Total (%)	*P* value
			<1000	1000–5000	>5000		
Sex							
Male	48 (24.0)	10 (20.8)	2 (4.2)	1 (2.1)	2 (4.2)	5 (10.4)	0.6713^a^
Female	152 (76.0)	27 (17.8)	7 (4.6)	6 (4.0)	5 (3.3)	15 (9.9)	0.705^b^
Age range							
<20	6 (3.0)	2 (33.3)	0 (0)	0 (0)	1 (16.7)	1 (16.7)	
21–30	55 (27.5)	12 (21.8)	3 (5.5)	1 (1.8)	1 (1.8)	5 (9.1)	
31–40	81 (40.5)	17 (21.0)	3 (3.7)	5 (6.2)	3 (3.7)	11 (13.6)	0.228^c^
41–50	40 (20.0)	6 (15.0)	1 (2.5)	1 (2.5)	1 (2.55)	3 (7.5)	
>50	18 (9.0)	0 (0)	0 (0)	0 (0)	0 (0)	0 (0)	0.638^d^

^a^Sex (examined number versus positive number); ^b^Sex (examined number versus parasite density); ^c^Age range versus positive number; ^d^Age range versus parasite density.

**Table 4:** CD4 count level and the prevalence of malaria among HIV patients.

CD4^+^ count	Frequency	Number with malaria parasitaemia (%)	*P* value
<200	45	7 (15.5)	0.011*
200–300	31	7 (22.6)	
351–500	70	6 (8.6)	
>500	54	17 (31.5)	
Total	200	37 (18.5)	

*Significant at <0.05.
